# Exploratory mRNA and microRNA profiling identifies transcriptomic alterations in the *PIK3CA*-related overgrowth spectrum

**DOI:** 10.3389/fgene.2026.1935186

**Published:** 2026-10-23

**Authors:** Tadashi Shiohama, Mami Uegami, Yoshitaka Kubota, Hirotomo Saitsu, Mitsuhiro Kato, Jun Matsushima, Daisuke Sawada, Shoko Yoshii, Nobuyuki Mitsukawa, Hiromichi Hamada

**Affiliations:** 1 Department of Pediatrics, Graduate School of Medicine, Chiba University, Chiba, Japan; 2 Department of Pediatrics, International University of Health and Welfare Narita Hospital, Narita, Japan; 3 Department of Neurology, Mayo Clinic, Rochester, MN, United States; 4 Department of Plastic, Reconstructive, and Aesthetic Surgery, Graduate School of Medicine, Chiba University, Chiba, Japan; 5 Department of Biochemistry, Hamamatsu University School of Medicine, Hamamatsu, Shizuoka, Japan; 6 Department of Pediatrics, Showa Medical University School of Medicine, Tokyo, Japan; 7 Department of Pathology, Graduate School of Medicine, Chiba University, Chiba, Japan; 8 Department of Pathology, Dokkyo Medical University Saitama Medical Center, Koshigaya, Japan

**Keywords:** fibroblast profiling, microRNA, mRNA, PIK3CA-related overgrowth spectrum (PROS), somatic mosaic mutation

## Abstract

**Background:**

*PIK3CA*-related overgrowth spectrum (PROS) is a group of disorders caused by somatic mosaic mutations in the *PIK3CA* gene, characterized by tissue overgrowth affecting multiple organs, including the skin, adipose tissue, blood vessels, bones, and nervous system. Although constitutive hyperactivation of PI3K-AKT-mTOR signaling in PROS promotes abnormal tissue development, remodeling, and segmental overgrowth, the downstream signaling networks are highly complex, and the precise molecular mechanisms underlying tissue overgrowth remain incompletely understood.

**Methods:**

In this exploratory study, we investigated molecular alterations associated with overgrowth in PROS by profiling mRNA and microRNA expression in fibroblasts derived from affected skin (pv-Fibro) and unaffected skin (non-pv-Fibro) from the same patient. Additionally, fibroblasts from three healthy individuals were pooled and analyzed as a single control sample.

**Results:**

We identified 24,034 human mRNAs using microarray-based gene expression profiling. Ten mRNAs were selected as candidate lesion-associated upregulated genes in pv-Fibro (≥5-fold vs. nc-Fibro and <2-fold change in non-pv-Fibro vs. nc-Fibro). In contrast, three mRNAs were selected as candidate lesion-associated downregulated genes in pv-Fibro (≤0.2-fold vs. nc-Fibro and >0.5-fold in non-pv-Fibro vs. nc-Fibro). For miRNA profiling, 4,603 human microRNAs were analyzed. hsa-miR-146a-5p was markedly upregulated in pv-Fibro (≥5-fold vs. nc-Fibro and <2-fold change in non-pv-Fibro vs. nc-Fibro), while hsa-miR-146b-5p was markedly downregulated (≤0.2-fold vs. nc-Fibro and >0.5-fold in non-pv-Fibro vs. nc-Fibro).

**Discussion:**

The observed changes in mRNA and miRNA expression represent candidate molecular alterations associated with inflammatory and tissue remodeling pathways in PROS pathology.

## 1 Introduction

### 1.1 Introduction

*PIK3CA*-related overgrowth spectrum (PROS) is a group of disorders caused by somatic mosaic mutations in the *PIK3CA* gene. This disease concept was proposed around 2013 and encompasses a range of overgrowth syndromes previously considered distinct entities (Keppler-Noreuil et al., 2015). Representative conditions include clinically heterogeneous disorders such as megalencephaly-capillary malformation (MCAP) (Mirzaa et al., 2012), congenital lipomatous overgrowth, vascular malformations, epidermal nevi, and scoliosis/skeletal anomalies (CLOVES) syndrome (Martinez-Lopez et al., 2017). This group of conditions is characterized by tissue overgrowth affecting multiple organs, including the skin, adipose tissue, blood vessels, bones, and the nervous system.

Recurrent activating *PIK3CA* missense variants identified in PROS include E542K and E545K in the helical domain and H1047R in the kinase domain of the p110α catalytic subunit (Pagliazzi et al., 2021). These mutations increase PI3Kα activity through distinct molecular mechanisms: helical domain mutations such as E542K and E545K disrupt the inhibitory interaction between the p110α helical domain and the p85α regulatory subunit, whereas the kinase domain mutation H1047R induces conformational changes that reduce p85α-mediated autoinhibition and enhance membrane-associated kinase activity. Consequently, these mutations increase phosphatidylinositol-3,4,5-trisphosphate (PIP3) production and subsequent activation of downstream AKT–mTOR signaling (Zhao and Vogt, 2008; Fruman et al., 2017; Liu et al., 2022). The presence of these activating mutations in a mosaic state during development is considered a major molecular basis of PROS, although the relationship between individual *PIK3CA* variants and specific clinical phenotypes is not absolute.

Although activating *PIK3CA* mutations are among the most common genetic alterations in human cancers (Wang et al., 2025), PROS is fundamentally distinct from neoplastic disorders. In PROS, postzygotic mosaic *PIK3CA* mutations arise during embryonic development and primarily promote tissue overgrowth, hypertrophy, and developmental dysregulation, rather than invasive growth and metastatic progression characteristic of malignant tumors. Importantly, the same activating *PIK3CA* variants can occur in both cancer and PROS, suggesting that the biological consequences of *PIK3CA* activation are strongly influenced by the developmental timing, mosaic distribution, and cellular and tissue context in which the mutation arises. Consequently, mosaic activation of the PI3K-AKT-mTOR pathway in PROS promotes abnormal tissue development, remodeling, and segmental overgrowth, rather than malignant transformation (Madsen et al., 2018). However, downstream signaling networks are highly complex, and the precise molecular mechanisms underlying tissue overgrowth remain unclear.

Historically, treatment primarily consisted of symptomatic interventions such as surgery and laser therapy. In recent years, PI3K inhibitors (Canaud et al., 2023), such as alpelisib, have emerged as promising molecularly targeted therapies for suppressing tissue overgrowth. Alpelisib targets PI3Kα, which is implicated in the pathogenesis of PROS, and has been reported to reduce lesion volume and improve pain and functional outcomes. However, the efficacy of PI3Kα inhibition for neurological manifestations of PROS remains incompletely defined, particularly with regard to neurodevelopmental delay, cognitive impairment, and epilepsy. Furthermore, although targeted PI3K inhibition can ameliorate several PROS-associated manifestations, the extent to which it can prevent or reverse established developmental abnormalities remains unclear.

This study aimed to explore molecular alterations associated with *PIK3CA* mosaicism in PROS by performing integrated mRNA and microRNA profiling of patient-derived fibroblasts. Previous studies on other disorders have demonstrated that transcriptomic and proteomic analyses of patient-derived fibroblasts can provide valuable insights into disease-associated molecular pathways (Shiohama et al., 2019). However, comprehensive expression profiling of PROS using fibroblasts has not been well established. In particular, the contribution of microRNA-mediated post-transcriptional regulation to the molecular phenotype of PROS remains poorly understood. Identifying disease-specific expression profiles through comparison with healthy controls is often complicated by the effects of sex, treatment history, and inter-individual variability, all of which may influence disease progression. This challenge is particularly pronounced when comparisons are made across different individuals. In contrast, comparison of *PIK3CA* mutation-positive and mutation-negative skin samples from the same patient enables an intra-individual comparison under a similar genetic and environmental background.

### 1.2 Case presentation

The proband was a Japanese patient who was born at term after a normal pregnancy and delivery and was followed up for macrocephaly with massive hyperostosis of the skull and psychomotor delay. Tonic seizures involving the right side of the body were noted on postnatal day 3. Magnetic resonance imaging (MRI) of the brain revealed left hemimegalencephaly. As the seizures were medically intractable, hemispherotomy was performed at 1 year and 7 months of age. Postoperatively, the seizures resolved and antiepileptic drugs were not required until seizure recurrence occurred at 21 years of age. At approximately 12 years of age, protrusion of the left frontal region became apparent and gradually progressed. At approximately 20 years of age, rapid enlargement of the left frontal region and facial overgrowth were observed, resulting in superior compression of the left globe (Figures 1A–C). Owing to concerns regarding ocular compression caused by progressive local tissue overgrowth, cranial reconstruction involving the frontal bone and zygoma was performed at our institution at 21 years of age. Histopathological findings of the lesional tissue were consistent with those of the epidermal nevus (Figure 1D). The lesion continued to show slow regrowth, and pain in the protruding left frontal region had developed since approximately 29 years of age; however, it was well controlled with analgesics. Although the left lower limb was slightly longer than the contralateral side, no apparent overgrowth was observed on the right side of the face, trunk, or extremities.

**FIGURE 1:**
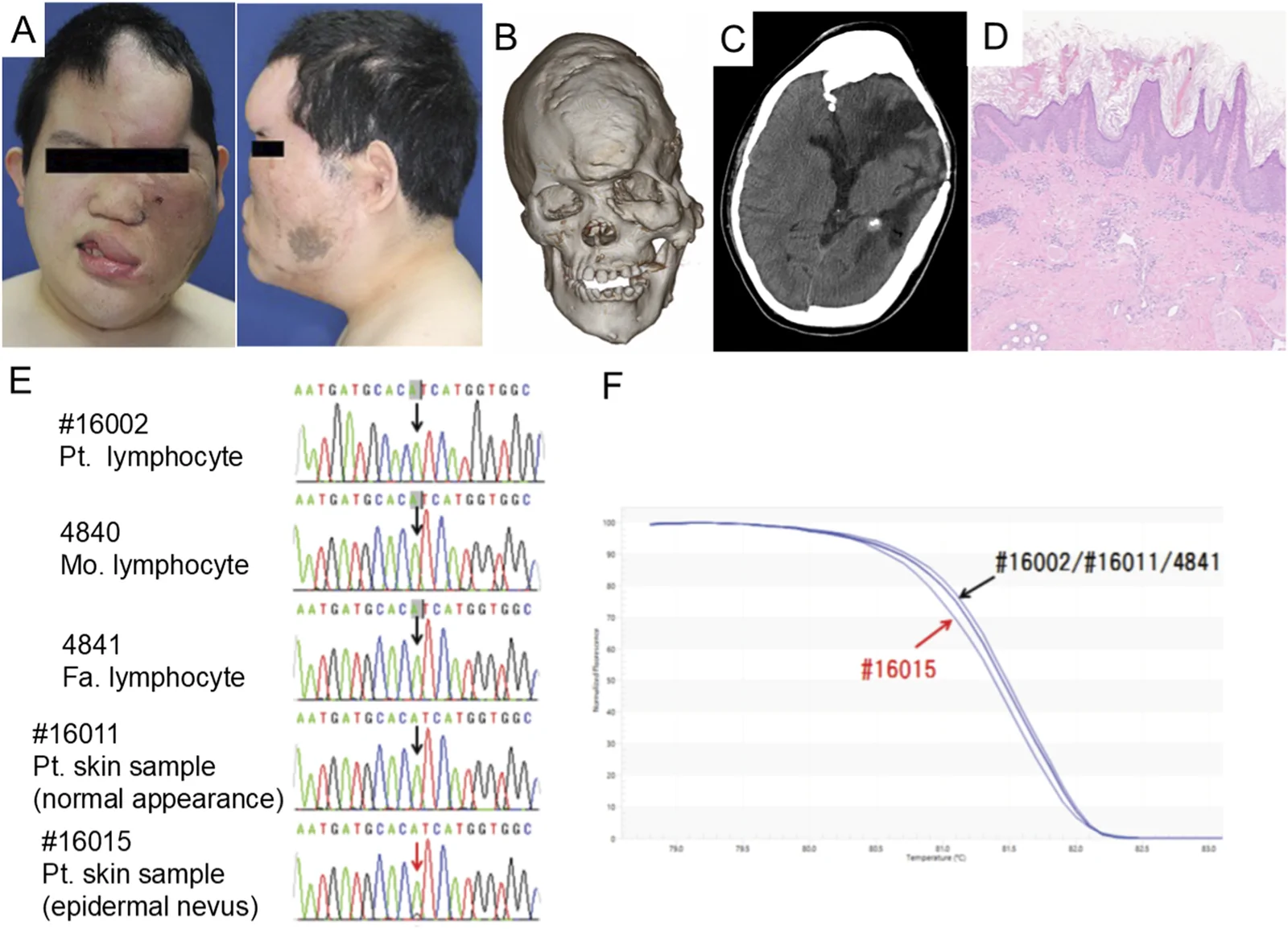
Facial enlargement centered on the left frontal region in a photograph **(A)**, three-dimensional facial CT **(B)**, and head CT **(C)** (preoperative at 21 years old). Epidermal nevus on Hematoxylin and eosin staining (x50) **(D)**. A *PIK3CA* mutation identified exclusively in the affected skin sample **(E)**. Abnormal melting curve with heteroduplex observed only in the affected skin samples **(F)**. Abbreviations: Fa., the patient’s father; Mo., the patient’s mother; Pt., the patient.

### 1.3 Genetic diagnosis

Whole exome sequencing (WES) was performed using DNA obtained from peripheral blood lymphocytes of the proband, his parents, and his affected and non-affected skin samples. A *PIK3CA* mutation (NM_006218.3:c.3140A>G, p.(His1047Arg), NC_000003.11:g.178952085A>G) was identified exclusively in the affected skin samples (Figure 1E). Furthermore, high-resolution melting (HRM) analysis was conducted using DNA derived from both affected and unaffected skin samples and the peripheral blood lymphocytes of the patient and his parents. An abnormal melting curve consistent with heteroduplex formation was observed only in affected skin samples. In contrast, identical melting profiles were observed in the unaffected skin and lymphocytes of the patient and his father (Figure 1F). Based on these findings, the patient was diagnosed with *PIK3CA*-related overgrowth spectrum (PROS) due to a somatic mosaic *PIK3CA* mutation.

## 2 Materials and methods

### 2.1 Human fibroblasts and cell lines

Skin fibroblasts were established from normal and affected skin in the case of PROS due to *PIK3CA* mosaicism (designated as non-pv-Fibro and pv-Fibro, respectively). The *PIK3CA* mosaic mutation was detected only in pv-Fibro cells. Normal control fibroblasts pooled from three healthy individuals were also analyzed. Normal human control fibroblasts (nc-Fibro) (N1#58732338, N2 #61447289, and N3 #61683453) were purchased from the American Type Culture Collection (ATCC, Manassas, VA, United States)^7^. Cells were cultured in DMEM supplemented with 10% fetal bovine serum and a 1% penicillin/streptomycin solution at 37 °C with 5% CO2. Cells were harvested, suspended in QIAzol Lysis Reagent (Qiagen, Hilden, Germany), and immediately stored at −80 °C for future experiments.

### 2.2 Microarray RNA preparation and quality assessment

Total RNA, including mRNA and microRNA, was extracted from cells using standard protocols. RNA quality and quantity were assessed using a spectrophotometer and Agilent 2,100 Bioanalyzer (Agilent Technologies, Santa Clara, CA, United States).

### 2.3 Human Gene 2.0 ST array expression analysis

A total of 500 ng of total RNA was used for cDNA synthesis and labeling. Fragmented and labeled cDNA samples were prepared using the GeneChip WT PLUS Reagent Kit (Affymetrix, CA, United States) according to the manufacturer’s instructions. A hybridization cocktail (total volume: 150 μL) was prepared, consisting of 109 μL Hybridization Master Mix and 3.5 μg of fragmented and labeled cDNA. Hybridization was performed using the GeneChip Hybridization Oven 645 (Affymetrix, CA, United States) at 45 °C for 16 h with rotation at 60 rpm. Following hybridization, arrays were washed and stained using the GeneChip Fluidics Station 450 (Affymetrix, CA, United States) according to the manufacturer’s protocol. Arrays were scanned using the GeneChip Scanner 3000 7G (Affymetrix, CA, United States), and image acquisition was performed according to the Affymetrix GeneChip Command Console (AGCC) version 4.0 user manual.

### 2.4 GeneChip miRNA 4.0 array expression analysis

A total of 1,000 ng of total RNA was used for labeling. Biotin-labeled samples were prepared using the FlashTag Biotin HSR RNA Labeling Kit (Affymetrix, CA, United States) according to the manufacturer’s instructions. A hybridization cocktail (total volume: 150 μL) was prepared, consisting of 110.5 μL Hybridization Master Mix and 21.5 μL of the biotin-labeled RNA sample. Hybridization was performed using the GeneChip Hybridization Oven 645 (Affymetrix, CA, United States) at 48 °C for 18 h with rotation at 60 rpm. After hybridization, arrays were washed and stained using the GeneChip Fluidics Station 450 (Affymetrix, CA, United States) according to the manufacturer’s instructions. Arrays were scanned using the GeneChip Scanner 3000 7G (Affymetrix, CA, United States), and image acquisition was performed according to the Affymetrix GeneChip Command Console (AGCC) version 4.0 user manual.

### 2.5 Data processing and statistical analysis

Raw intensity data from the Affymetrix GeneChip Human Gene 2.0 ST Array and GeneChip miRNA 4.0 Array were processed using Affymetrix Transcriptome Analysis Console (TAC) software. Background correction, normalization, and probe summarization were performed using the robust multiarray average (RMA) algorithm. Probe sets with consistently low signal intensities across all samples were excluded prior to downstream analysis to reduce noise and improve data reliability. Differential gene expression was evaluated using fold-change comparisons between groups. Genes showing ≥5-fold increase or ≤0.2-fold decrease in pv-Fibro compared with nc-Fibro were defined as markedly upregulated or downregulated, respectively. To identify lesion-associated changes in expression, additional filtering was applied using a non-pv-Fibro vs. nc-Fibro comparison. Genes showing a <2-fold change in non-pv-Fibro relative to nc-Fibro were considered lesion-associated upregulated candidates, whereas genes showing a >0.5-fold change in non-pv-Fibro relative to nc-Fibro were considered lesion-associated downregulated candidates. The same preprocessing and fold-change-based criteria were applied to miRNA expression data. Functional enrichment analysis was performed using the STRING database. Statistical significance for enrichment analysis was defined as *p* < 0.05. No formal statistical testing was performed for differential gene expression; it was assessed solely using fold-change thresholds. Normal control fibroblasts derived from three healthy individuals were pooled and analyzed as a single control sample. All analyses were performed using the standard workflow in the TAC software, according to the manufacturer’s instructions, without modifying the default parameters.

## 3 Results

### 3.1 mRNA and microRNA profiling in PROS and NC fibroblasts

To identify the mRNAs and miRNAs involved in the pathogenesis of PROS associated with *PIK3CA* mosaicism, we performed mRNA and miRNA expression profiling of pooled samples of affected skin-derived fibroblasts (pv-Fibro), unaffected skin-derived fibroblasts (non-pv-Fibro), and three normal control fibroblast lines (nc-Fibro) using whole-transcriptome expression microarray and miRNA array analyses.

Of the 24,034 human mRNAs, 17,599 were selected for further analysis after filtering out xhyb. When pv-Fibro was compared with nc-Fibro, 38 genes were upregulated (≥5-fold change), and 36 genes were downregulated (≤0.2-fold change) (Supplementary Table S1). Gene enrichment analysis of the upregulated genes revealed significant enrichment in biological processes, including extracellular matrix organization, regulation of systemic processes, regulation of cellular response to growth factor stimuli, axon guidance, cell–cell adhesion, and myometrial relaxation and contraction pathways (Figure 2A). In addition, STRING analysis indicated enrichment in the Gene Ontology (GO) term “collagen-containing extracellular matrix.” Among the 407 genes annotated in the STRING database for this GO term, 7 of the 38 input genes were mapped with a statistically significant enrichment (*p* = 0.0172) (Figure 2B). For the downregulated genes, gene enrichment analysis identified pathways including ABC transporters in lipid homeostasis, regulation of blood circulation, NABA ECM glycoproteins, negative regulation of cell migration, tube morphogenesis, ncRNAs in Wnt signaling in hepatocellular carcinoma, and regulation of osteoblast differentiation (Figure 2A). An enrichment related to ABC transporter-associated functions was also observed. STRING analysis further supported this enrichment, with 3 of the 36 downregulated genes mapping to the corresponding ABC transporter-related category (p = 0.0306) (Figure 2B). This finding represents a pathway-level enrichment, and its biological significance requires further investigation. To extract lesion-associated gene expression signatures, we further filtered genes that were upregulated (>5-fold) in pv-Fibro compared with nc-Fibro but showed minimal changes (<2-fold change) in non-pv-Fibro compared with nc-Fibro. This analysis identified ten mRNAs (*WFDC1, L1CAM, MX1, KCNMB1, OLFM2, MYH11, CORIN, DOK5, EPHA5*, and *MT1F*) (Table 1). Similarly, among the genes downregulated (<0.2-fold change) in pv-Fibro versus nc-Fibro, those showing relatively stable expression (>0.5-fold change) in non-pv-Fibro versus nc-Fibro were selected, resulting in the identification of three mRNAs (*SFRP1, HOXB3*, and *NRN1*) as lesion-associated downregulated genes (Table 1).

**FIGURE 2:**
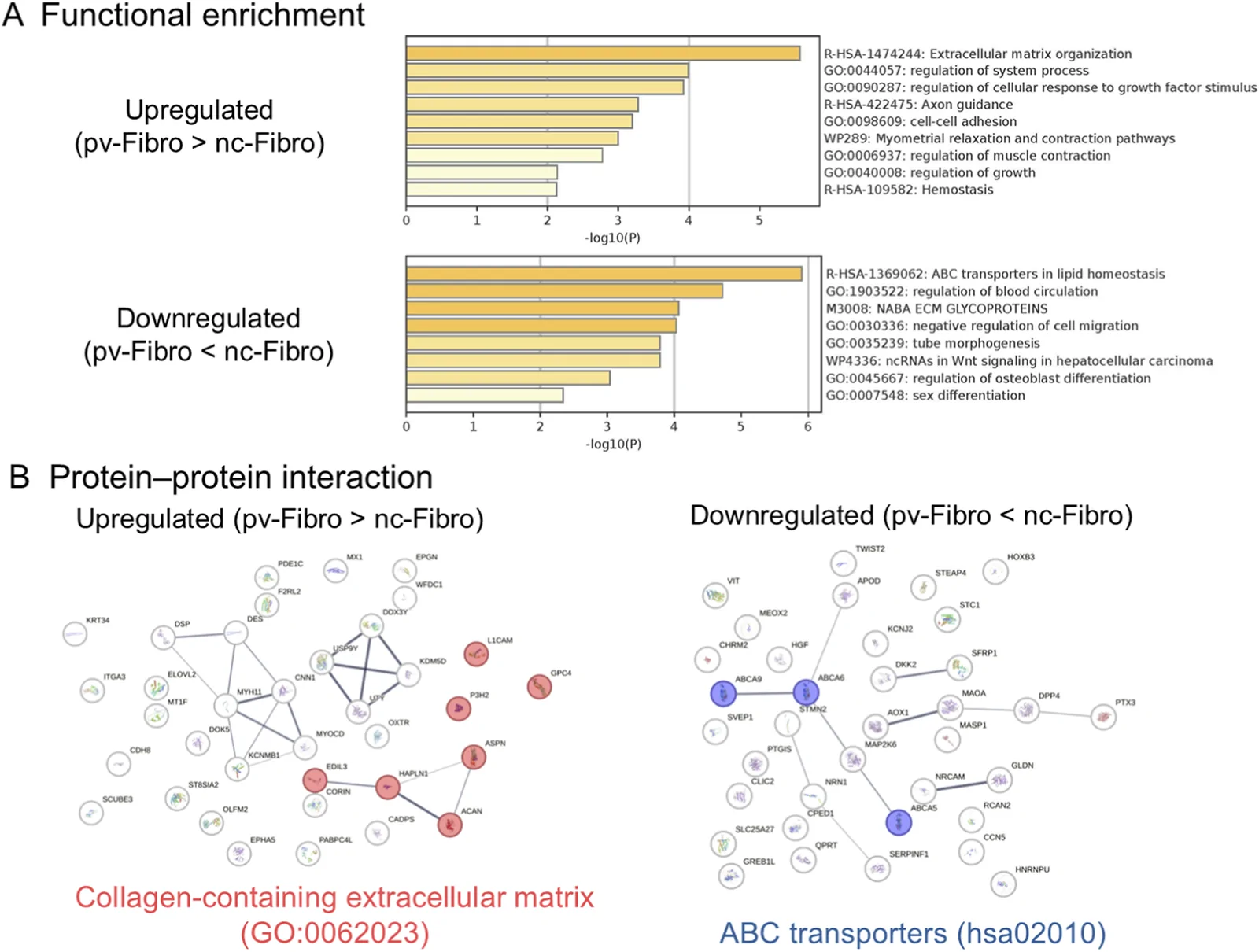
Functional enrichment and protein–protein interaction analyses of DEGs. Metascape analysis identified significantly enriched GO terms and pathways among upregulated and downregulated mRNAs **(A)**. STRING-generated PPI networks revealed enrichment of “collagen-containing extracellular matrix” in upregulated genes and ABC transporters in downregulated genes **(B)**.

**TABLE 1:** The list of mRNAs with >5-fold change in expression.

Gene symbol	Chr	Expression values of nc-Fibro	Expression values of non-pv-Fibro	Expression values of pv-Fibro	Expression rates of pv-Fibro per nc-Fibro	Expression rates of non-pv-Fibro per nc-Fibro
*Upregulated mRNA* (*N* = 10)						
*WFDC1*	16	39.6	46.0	713.1	18.0	1.2
*L1CAM*	X	17.3	16.5	175.1	10.1	0.95
*MX1*	21	16.5	20.1	111.9	6.8	1.2
*KCNMB1*	5	17.2	24.5	112.0	6.5	1.4
*OLFM2*	19	88.5	89.7	553.4	6.3	1.0
*MYH11*	16	27.2	27.4	163.2	6.0	1.0
*CORIN*	4	24.7	20.2	146.9	5.9	0.81
*DOK5*	20	42.1	72.7	233.6	5.6	1.7
*EPHA5*	4	10.2	18.4	52.7	5.2	1.8
*MT1F*	16	24.7	17.7	126.1	5.1	0.72
*Downregulated mRNA* (*N* = 3)						
*SFRP1*	8	237.2	158.2	37.6	0.16	0.67
*HOXB3*	17	107.3	58.3	17.7	0.17	0.54
*NRN1*	6	1840.1	2025.8	346.1	0.19	1.1

Abbreviation: Chr, chromosome; Abbreviation: nc-Fibro, three normal control fibroblast lines; non-pv-Fibro, unaffected skin-derived fibroblasts; pv-Fibro, affected skin-derived fibroblasts.

In total, 4,603 human miRNAs were examined (Supplementary Table S1). Compared to nc-Fibro, six miRNAs were upregulated (≥ 5-fold change), and four miRNAs were downregulated (≤ 0.2-fold change) in pv-Fibro (Table 2; Supplementary Table S3). After applying the same filtering criteria using non-pv-Fibro versus nc-Fibro comparisons (<2-fold change for upregulated candidates and >0.5-fold change for downregulated candidates), two lesion-associated miRNAs were identified: hsa-miR-146a-5p and hsa-miR-146b-5p (Table 2; Supplementary Table S3). These findings revealed candidate lesion-associated alterations in mRNA and miRNA expression in PROS fibroblasts, including genes associated with inflammatory and extracellular matrix-related pathways.

**TABLE 2:** The list of microRNAs with >5-fold change in expression.

microRNA	Expression values of nc-Fibro	Expression values of non-pv-Fibro	Expression values of pv-Fibro	Expression rates of pv-Fibro per nc-Fibro	Expression rates of non-pv-Fibro per nc-Fibro	Candidate microRNAs
Upregulated microRNA (N = 6)						
hsa-miR-412-5p	2.5	17.6	27.3	11.1	7.2	​
hsa-miR-3935	3.7	36.6	39.5	10.5	9.8	​
hsa-miR-6780b-5p	9.4	91.5	81.7	8.7	9.7	​
hsa-miR-4304	3.7	7.6	27.1	7.2	2.0	​
hsa-miR-146a-5p	21.4	20.8	127.3	6.0	0.97	✓
hsa-miR-501-5p	5.9	12.6	33.9	5.8	2.14	​
Downregulated microRNA (N = 4)						
hsa-miR-10a-5p	851.4	240.6	4.0	0.0047	0.28	​
hsa-miR-3178	4,772.7	232.3	394.9	0.083	0.049	​
hsa-miR-3180	128.6	8.6	12.4	0.097	0.067	​
hsa-miR-146b-5p	23.5	27.8	3.9	0.17	1.2	✓

Check indicates the candidate microRNAs. Abbreviation: nc-Fibro, three normal control fibroblast lines; non-pv-Fibro, unaffected skin-derived fibroblasts; pv-Fibro, affected skin-derived fibroblasts

### 3.2 Predictive target genes of miR-146a-5p and miR-146b-5p

We used the miRDB (http://mirdb.org) *in silico* database to identify 30 predicted target genes of hsa-miR-146a-5p with target scores greater than 90 (Supplementary Table S4). According to publicly available databases, four of these genes (*WWC2*, *NUMB*, *CARD10*, and *ZNF540*) are expressed in fibroblast tissues (The Human Protein Atlas, http://www.proteinatlas.org/). Consistently, the same four genes (*WWC2*, *NUMB*, *CARD10*, and *ZNF540*) were predicted to be hsa-miR-146a-5p targets with a score greater than 90 using miRDB (Supplementary Table S4). However, none of these predicted target genes overlapped with the mRNAs identified in our analysis. Although DOK5 had a relatively low target score (52), it was identified as a predicted target of hsa-miR-146b-5p by miRDB. To further assess the predicted targets using an independent prediction algorithm, we examined the target genes of hsa-miR-146a-5p and hsa-miR-146b-5p using TargetScanHuman version 8.0 (https://www.targetscan.org/vert_80/, access on 27 August 2026) (McGeary et al., 2019). None of the 10 upregulated or three downregulated lesion-associated mRNAs identified in our analysis were predicted as targets of the corresponding miRNAs (Supplementary Table S5). These findings indicate that no direct correspondence between the identified lesion-associated mRNAs and the computationally predicted targets of hsa-miR-146a-5p or hsa-miR-146b-5p could be established in the present dataset.

### 3.3 Histopathological findings and molecular background

The histopathological findings of the lesional tissue from which the pv-Fibro was established were consistent with those of the epidermal nevus (Figure 1D). A subset of epidermal nevi arises from somatic mosaic mutations in the *PIK3CA* gene and is considered a cutaneous manifestation within the clinical spectrum of *PIK3CA*-related overgrowth spectrum (PROS).

## 4 Discussion

In this study, we performed paired transcriptomic profiling of affected and unaffected fibroblasts derived from a patient with PROS carrying a somatic mosaic *PIK3CA* mutation. This approach enabled us to identify lesion-associated transcriptional alterations while minimizing inter-individual variability. The observed transcriptional changes primarily involved pathways related to extracellular matrix organization, growth factor signaling, cell adhesion, vascular and smooth muscle-related functions, and inflammatory responses. Functional enrichment analysis indicated that the upregulated mRNAs were predominantly associated with extracellular matrix organization, growth factor signaling, cell adhesion, and smooth muscle–related pathways. These findings were further supported by interaction network analysis, which showed a significant enrichment of collagen-containing extracellular matrix components, suggesting activation of tissue remodeling processes.

Conversely, for the downregulated mRNAs, functional enrichment analysis indicated enrichment in pathways related to lipid transport, vascular regulation, and tube morphogenesis, and network analysis suggested involvement of ABC transporter-associated functions. ABC transporters have diverse physiological and pathological functions, including roles in lipid transport and cellular homeostasis. Because disease controls were not included, the biological and disease specificity of this enrichment remains uncertain. Therefore, the ABC transporter enrichment observed in the present study should be interpreted as a context-dependent transcriptional feature associated with the *PIK3CA*-mosaic lesion rather than as a PROS-specific molecular signature.

Although PROS is rare, large-scale proteomic and transcriptomic analyses comparable to those conducted in cancer research remain limited. Previous studies of PROS have primarily relied on targeted genomic analyses and the detection of low-frequency mosaic variants (Loconte et al., 2015), with a major focus on elucidating mechanisms underlying PI3K-AKT-mTOR pathway activation (Keppler-Noreuil et al., 2015; Rivière et al., 2012; Kurek et al., 2012). Recently, single-cell RNA sequencing approaches have been introduced, enabling the investigation of cellular heterogeneity and mutation-specific transcriptional programs within affected tissues. Wedemeyer et al. performed single-cell long-read RNA sequencing of lesions from patients with MCAP and demonstrated that *PIK3CA*-mutant cells were enriched in a *PAX3*-positive fibroblast-like population. These cells exhibited increased expression of *PAX3, SOX11, CXCL14, NCAM1*, and *TUBB2B*, together with decreased expression of *NOTCH3* and *HES1*, suggesting an immature or developmental mesenchymal transcriptional program (Wedemeyer et al., 2024). Although both the present study and that of Wedemeyer et al. investigated *PIK3CA*-related disorders, direct concordance in gene expression profiles was not expected due to differences in the tissues analyzed and the resolution of the analyses. By analyzing PROS lesions at the tissue level, our study provides an exploratory molecular profile of the affected fibroblasts that reflects molecular alterations associated with the affected tissue.

Transcriptomic studies in cancer biology have shown that *PIK3CA* activation induces broad alterations in gene regulation, including transcriptional and splicing programs (Ladewig et al., 2022). Despite these advances, integrated analyses that incorporate miRNA expression in PROS tissues remain scarce.

In this study, to enrich for lesion-associated transcriptional alterations and minimize changes unrelated to the *PIK3CA*-mutant lesion, we further filtered the upregulated genes by selecting those showing less than a 2-fold change in non-pv-Fibro compared to nc-Fibro. This filtering yielded ten mRNAs: *WFDC1*, *L1CAM*, *MX1*, *KCNMB1*, *OLFM2*, *MYH11*, *CORIN*, *DOK5*, *EPHA5*, and *MT1F*. The upregulated mRNAs can be broadly classified into four functional groups. Notably, several genes appeared in multiple functional categories, reflecting their overlapping biological roles.

Several genes are associated with tissue organization, ECM remodeling, and cell–cell communication. *WFDC1, L1CAM*, and *EPHA5* form a functionally related group involved in regulating ECM organization, cell adhesion, and contact-dependent signaling. *WFDC1* is a secreted protein that contributes to ECM maintenance, preservation of tissue structure, and the suppression of excessive inflammatory responses (Ressler et al., 2014). Reduced *WFDC1* expression has been linked to tumorigenesis, including prostate cancer, suggesting its potential tumor-suppressive role (Ressler and Rowley, 2011). *L1CAM* mediates both homophilic and heterophilic cell adhesion by interacting with integrins, extracellular matrix components, and other cell surface receptors, thereby regulating cell adhesion, migration, and axonal outgrowth (Raveh et al., 2009). *EPHA5* is a receptor tyrosine kinase that interacts with membrane-bound ephrin ligands to regulate the adhesion and repulsion signals involved in tissue boundary formation and spatial organization (Kania and Klein, 2016). Collectively, these genes suggest activation of pathways that coordinate tissue architecture and cellular positioning.

Second, a subset of genes is associated with developmental and neuronal signaling pathways. *L1CAM* (Linneberg et al., 2019), *DOK5* (Shi et al., 2006), and *EPHA5* (Das et al., 2016) have been implicated in nervous system development and neuronal differentiation. These findings suggest that developmental signaling programs may be altered or represented in the transcriptional profile of affected fibroblasts.

Third, genes related to inflammatory responses, stress adaptation, and metabolic homeostasis were upregulated. *MX1* and *MT1F* are associated with interferon signaling, oxidative stress responses, and cellular defense mechanisms. *MX1* is involved in antiviral and autoimmune responses (Jung et al., 2019), whereas *MT1F* protects against oxidative stress and regulates metal homeostasis (Ruttkay-Nedecky et al., 2013). *WFDC1* may partially counterbalance these inflammatory responses through its anti-inflammatory properties (Ressler et al., 2014).

Additionally, *OLFM2* and *CORIN* are linked to metabolic and homeostatic regulation. *OLFM2* has been implicated in adipocyte differentiation, metabolism, and cell cycle regulation (Lluch et al., 2025). In contrast, *CORIN* regulates blood pressure and fluid balance by activating atrial natriuretic peptide (ANP) (Gong et al., 2025). The upregulation of these genes may reflect adaptive metabolic and homeostatic responses within the affected tissues.

Finally, several genes are associated with vascular and smooth muscle–related functions. *KCNMB1, MYH11,* and *CORIN* are involved in vascular smooth muscle contractility, vascular tone regulation, and circulatory homeostasis. Their coordinated upregulation suggests vascular remodeling and altered local hemodynamic regulation. Overall, these coordinated transcriptional changes suggested the involvement of interconnected pathways related to tissue remodeling, developmental signaling, inflammatory and stress responses, metabolic adaptation, and vascular homeostasis in pv-Fibro cells.

Conversely, among the genes downregulated (≤0.2-fold) in pv-Fibro compared with nc-Fibro, we aimed to identify lesion-associated local downregulation by excluding genes that showed substantial changes in non-pv-Fibro. Specifically, we removed genes with a fold change of ≤0.5 in non-pv-Fibro relative to nc-Fibro. This filtering strategy identified three mRNAs, *SFRP1*, *HOXB3*, and *NRN1*. *SFRP1* is an inhibitor of Wnt signaling, and its downregulation has been associated with altered Wnt signaling and tumorigenesis in other disease contexts, raising the possibility that Wnt-related signaling may be altered in the affected fibroblasts (Baharudin et al., 2020). *HOXB3* is a homeobox transcription factor involved in the embryonic patterning and developmental regulation of neural crest- and mesoderm-derived tissues. Therefore, reduced *HOXB3* expression may reflect altered developmental programs associated with *PIK3CA*-driven tissue overgrowth (Nelms and Labosky, 2010). *NRN1* is a neurotrophic molecule primarily involved in neuronal development and synaptic plasticity; however, its role in fibroblasts remains unclear.

To identify lesion-associated miRNA alterations, the same filtering strategy used for the mRNA analysis was applied. Among miRNAs showing marked differential expression in pv-Fibro compared with nc-Fibro (≥5-fold increase or ≤0.2-fold decrease), those exhibiting minimal expression changes in non-pv-Fibro compared with nc-Fibro (<2-fold for upregulated candidates and >0.5-fold for downregulated candidates) were selected. This analysis identified two lesion-associated miRNAs, hsa-miR-146a-5p and hsa-miR-146b-5p. Hsa-miR-146a-5p was upregulated, whereas hsa-miR-146b-5p was downregulated. The miR-146 family is known to negatively regulate NF-κB signaling by targeting *TRAF6* and *IRAK1*, thereby functioning as feedback regulators of innate immune and inflammatory responses (Paterson and Kriegel, 2017). The differential expression of miR-146 family members may reflect altered inflammatory signaling in PROS fibroblasts. However, because NF-κB activity was not directly assessed and no functional experiments were performed, the present findings do not establish a causal relationship between miR-146a/b expression and inflammatory pathway activation. Further experimental studies will be required to determine the biological significance of these miRNA alterations in PROS. Although miR-146a-5p and miR-146b-5p share highly similar seed sequences and target genes, they are encoded by distinct genomic loci and are subject to different transcriptional regulation, warranting an isoform-specific interpretation (Paterson and Kriegel, 2017; Mirzaei et al., 2024).

Currently, treatment for PROS is primarily symptomatic; however, molecularly targeted therapies, including mTOR inhibitors and PI3K-selective inhibitors such as alpelisib (Canaud et al., 2023), have been introduced into clinical practice. Although not specific to PROS, emerging therapeutic strategies targeting dysregulated miRNAs (e.g., the miR-146 family) are under investigation (Mirzaei et al., 2024). Moreover, potential dysregulation of Wnt signaling, together with extracellular matrix and vascular remodeling, may represent pathways for future investigation in PROS. Taken together, these findings provide candidate molecular pathways for further investigation rather than established therapeutic targets for PROS. Based on these findings, we propose a conceptual model linking somatic *PIK3CA* mutation with the transcriptomic alterations observed in PROS fibroblasts (Supplementary Figure S1).

This study has several limitations. First, this study included samples from only a single patient, which substantially limits the generalizability of the findings and precludes assessment of inter-individual variability. In addition, fibroblasts from three healthy individuals were pooled and analyzed as a single control sample, preventing estimation of biological variability among healthy controls and precluding conventional statistical comparisons across independent biological replicates. Therefore, the fold-change-based filtering strategy used in this study should be regarded as an exploratory approach for identifying candidate lesion-associated alterations rather than as a formal differential expression analysis. Nevertheless, the comparison of genetically affected and unaffected fibroblasts from the same patient provides an important intra-individual control that minimizes some confounding effects related to genetic background, sex, and environmental exposure. Second, the present study was performed using dermal fibroblasts, which cannot fully recapitulate the cellular heterogeneity, tissue architecture, and intercellular interactions of PROS lesions *in vivo*. Because PROS can affect multiple tissues with distinct developmental and functional characteristics, the molecular alterations identified in fibroblasts may not be representative of other affected tissues. In addition, although ABC transporter-associated functions were enriched among the downregulated genes, the present study did not determine whether this alteration is specific to PROS or shared with other pathological conditions, including cancer. Comparative analyses using independent PROS cohorts and appropriate disease controls will be necessary to establish the specificity and biological significance of this finding. Third, in this study, known targets of miR-146a/b were identified using miRTarBase 2.0. However, no direct associations were identified with the upregulated mRNAs, and only *SFRP1*, which was downregulated, was identified as a previously reported target of miR-146a. Furthermore, although DOK5 was predicted as a target of hsa-miR-146b-5p by miRDB with a relatively low target score (52), none of the lesion-associated differentially expressed mRNAs were predicted as targets by TargetScanHuman. This limited concordance may reflect differences between computational target prediction and the actual regulatory context of miRNAs in fibroblasts. Because experimental validation was not performed, the differential expression of the key candidate molecules identified in this study, including *WFDC1*, *SFRP1*, hsa-miR-146a-5p, and hsa-miR-146b-5p, could not be independently confirmed by RT-qPCR or other experimental approaches. In addition, functional assays were not performed to determine whether the identified miRNAs directly regulate candidate target genes. Therefore, these molecules should be regarded as exploratory candidates rather than validated molecular markers or therapeutic targets. Independent expression validation in additional PROS samples and functional studies, including miRNA–target interaction assays where feasible, will be necessary before further mechanistic conclusions can be drawn. Overall, the present findings should be considered exploratory and hypothesis-generating given the single-patient design, pooled control analysis, and lack of functional validation. Validation in additional PROS cases and mechanistic studies will be necessary to determine whether the identified transcriptomic alterations are reproducible and directly contribute to disease pathogenesis.

In conclusion, this study identified disease-associated mRNA and miRNA changes in expression in the PIK3CA-related overgrowth spectrum (PROS) using patient-derived fibroblasts. These results suggest that the activation of inflammatory, tissue remodeling, and developmental signaling pathways may contribute to disease pathology. Although limited by a single case, fibroblast-based analysis, and a lack of functional validation, these findings provide preliminary insights into the molecular landscape of PROS. They may help identify potential targets for future investigations. Further studies in additional PROS cases and experimental model systems are required to validate these findings and clarify their contribution to disease pathogenesis. These results provide a foundation for future investigations of the molecular mechanisms underlying tissue overgrowth in PROS.

## Data Availability

The original contributions presented in the study are publicly available. The dataset can be accessed in the GEO repository with the accession numbers GSE349641 and GSE349642.
