# Supplementary material for: Exploratory mRNA and microRNA profiling identifies transcriptomic alterations in the PIK3CA-related overgrowth spectrum
Source: Front Genet. 2026 Oct 23;17:1935186. doi: 10.3389/fgene.2026.1935186 (PMC13739472; doi:10.3389/fgene.2026.1935186)
Supplement: Supplementary file 1 [file Table3.docx]

| **Table S3**  The list of miRNA with over five-fold change in expression | | | |
| --- | --- | --- | --- |
| microRNA | Chr | Length | Sequence |
| *Upregulated miRNA* (*N*=6) | | |  |
| hsa-miR-412-5p | 14 | 23 | UGGUCGACCAGUUGGAAAGUAAU |
| hsa-miR-3935 | 16 | 22 | UGUAGAUACGAGCACCAGCCAC |
| hsa-miR-6780b-5p | 6 | 23 | UGGGGAAGGCUUGGCAGGGAAGA |
| hsa-miR-4304 | 12 | 17 | CCGGCAUGUCCAGGGCA |
| hsa-miR-146a-5p | 5 | 22 | UGAGAACUGAAUUCCAUGGGUU |
| hsa-miR-501-5p | X | 22 | AAUCCUUUGUCCCUGGGUGAGA |
| *Downregulated miRNA* (*N*=4) | | |  |
| hsa-miR-10a-5p | 17 | 23 | UACCCUGUAGAUCCGAAUUUGUG |
| hsa-miR-3178 | 16 | 17 | GGGGCGCGGCCGGAUCG |
| hsa-miR-3180 | 16 | 19 | UGGGGCGGAGCUUCCGGAG |
| hsa-miR-146b-5p | 10 | 22 | UGAGAACUGAAUUCCAUAGGCU |
