# Supplementary material for: Exploratory mRNA and microRNA profiling identifies transcriptomic alterations in the PIK3CA-related overgrowth spectrum
Source: Front Genet. 2026 Oct 23;17:1935186. doi: 10.3389/fgene.2026.1935186 (PMC13739472; doi:10.3389/fgene.2026.1935186)
Supplement: Supplementary file 2 [file Table4.docx]

| **Table S4**  The list of top target genes of hsa-miR-146a-5p and has miR-146b-5p | | | | | | | | |
| --- | --- | --- | --- | --- | --- | --- | --- | --- |
| **hsa-miR-146a-5p target genes** | | | | | **hsa-miR-146b-5p target genes** | | | |
| Target Rank | Target Score | Gene Symbol | High expression in fibroblast | | Target Rank | Target Score | Gene Symbol | High expression in fibroblast |
| 1 | 100 | TRAF6 |  |  | 1 | 100 | TRAF6 |  |
| 2 | 100 | IRAK1 |  |  | 2 | 99 | IRAK1 |  |
| 3 | 99 | SEC23IP |  |  | 3 | 99 | SEC23IP |  |
| 4 | 98 | NOVA1 |  |  | 4 | 98 | NOVA1 |  |
| 5 | 97 | PPP1R11 |  |  | 5 | 97 | UPP2 |  |
| 6 | 97 | UPP2 |  |  | 6 | 97 | WWC2 | ✓ |
| 7 | 97 | WWC2 | ✓ |  | 7 | 97 | PPP1R11 |  |
| 8 | 96 | BCORL1 |  |  | 8 | 96 | BCORL1 |  |
| 9 | 95 | ZNF649 |  |  | 9 | 95 | SORT1 |  |
| 10 | 95 | SORT1 |  |  | 10 | 95 | ZNF649 |  |
| 11 | 94 | NUMB | ✓ |  | 11 | 94 | NUMB | ✓ |
| 12 | 94 | USP32 |  |  | 12 | 94 | USP32 |  |
| 13 | 94 | PLSCR4 |  |  | 13 | 94 | PLSCR4 |  |
| 14 | 93 | CARD10 | ✓ |  | 14 | 93 | GDAP1L1 |  |
| 15 | 93 | SRSF6 |  |  | 15 | 93 | SIAH2 |  |
| 16 | 93 | APPL1 |  |  | 16 | 93 | SRSF6 |  |
| 17 | 93 | ZNF652 |  |  | 17 | 93 | APPL1 |  |
| 18 | 93 | SIAH2 |  |  | 18 | 93 | ZNF652 |  |
| 19 | 93 | GDAP1L1 |  |  | 19 | 93 | CARD10 | ✓ |
| 20 | 92 | SLC10A3 |  |  | 20 | 92 | MRS2 |  |
| 21 | 92 | MRS2 |  |  | 21 | 92 | EIF4G2 |  |
| 22 | 92 | ZNF354B |  |  | 22 | 92 | ZNF540 | ✓ |
| 23 | 92 | C9orf72 |  |  | 23 | 92 | SLC10A3 |  |
| 24 | 92 | EIF4G2 |  |  | 24 | 92 | C9orf72 |  |
| 25 | 92 | ZNF540 | ✓ |  | 25 | 92 | ZNF354B |  |
| 26 | 92 | GRIA3 |  |  | 26 | 92 | GRIA3 |  |
| 27 | 91 | LOC388813 | |  | 27 | 91 | DDHD1 |  |
| 28 | 91 | DDHD1 |  |  | 28 | 91 | LOC388813 | |
| 29 | 91 | BRK1 |  |  | 29 | 91 | TDRKH |  |
| 30 | 91 | TDRKH |  |  | 30 | 91 | BRK1 |  |
